# Re-Directing CD4^+^ T Cell Responses with the Flanking Residues of MHC Class II-Bound Peptides: The Core is Not Enough

**DOI:** 10.3389/fimmu.2013.00172

**Published:** 2013-07-01

**Authors:** Christopher J. Holland, David K. Cole, Andrew Godkin

**Affiliations:** ^1^Institute of Infection and Immunity, Cardiff University School of Medicine, Cardiff, UK; ^2^Department of Integrated Medicine, University Hospital of Wales, Cardiff, UK

**Keywords:** modified peptide, peptide flanking residue, peptide-major histocompatibility complex class II, T cell receptor, T cell repertoire, vaccine, crystal structure, MHC processing

## Abstract

Recombinant αβ T cell receptors, expressed on T cell membranes, recognize short peptides presented at the cell surface in complex with MHC molecules. There are two main subsets of αβ T cells: CD8^+^ T cells that recognize mainly cytosol-derived peptides in the context of MHC class I (pMHC-I), and CD4^+^ T cells that recognize peptides usually derived from exogenous proteins presented by MHC class II (pMHC-II). Unlike the more uniform peptide lengths (usually 8–13mers) bound in the MHC-I closed groove, MHC-II presented peptides are of a highly variable length. The bound peptides consist of a core bound 9mer (reflecting the binding motif for the particular MHC-II type) but with variable peptide flanking residues (PFRs) that can extend from both the N- and C-terminus of the MHC-II binding groove. Although pMHC-I and pMHC-II play a virtually identical role during T cell responses (T cell antigen presentation) and are very similar in overall conformation, there exist a number of subtle but important differences that may govern the functional dichotomy observed between CD8^+^ and CD4^+^ T cells. Here, we provide an overview of the impact of structural differences between pMHC-I and pMHC-II and the molecular interactions with the T cell receptor including the functional importance of MHC-II PFRs. We consider how factors such as anatomical location, inflammatory milieu, and particular types of antigen presenting cell might, in theory, contribute to the quantitative (i.e., pMHC ligand frequency) as well as qualitative (i.e., variable PFR) nature of peptide epitopes, and hence offer a means of control and influence of a CD4^+^ T cell response. Lastly, we review our recent findings showing how modifications to MHC-II PFRs can modify CD4^+^ T cell antigen recognition. These findings may have novel applications for the development of CD4^+^ T cell peptide vaccines and diagnostics.

## Introduction

T cell immunity is mediated primarily by the membrane bound T cell receptor (TCR) that interacts with peptide epitopes presented by major histocompatibility molecules (pMHC) (1). This interaction governs T cell specificity and leads to downstream T cell activation. Classical MHC exists in two forms: MHC class I (MHC-I) and MHC class II (MHC-II), which differ in both their subunit composition and functional expression pattern. MHC-I presents peptides derived mainly from endogenous cytosolic proteins and is expressed upon the cell surface of most nucleated cells allowing cognate CD8^+^ T cells to scan cells for intracellular infections or abnormal proteins in cancerous cells (2, 3). In contrast, MHC-II is expressed mainly upon antigen presenting cells (APCs) e.g., dendritic cells and macrophages, that patrol the extracellular space, actively endocytosing potentially immunogenic proteins that are proteolysed and complexed with MHC-II (pMHC-II). Activated APCs enter the lymphatic system and travel to secondary lymphoid nodes allowing naive CD4^+^ T cells to interrogate cell surface expressed pMHC-II enabling CD4^+^ T cell activation and initiation of immune responses (3–5).

## Peptides Presented by MHC-I and MHC-II have Distinct Structural Characteristics

**Figure 1:**
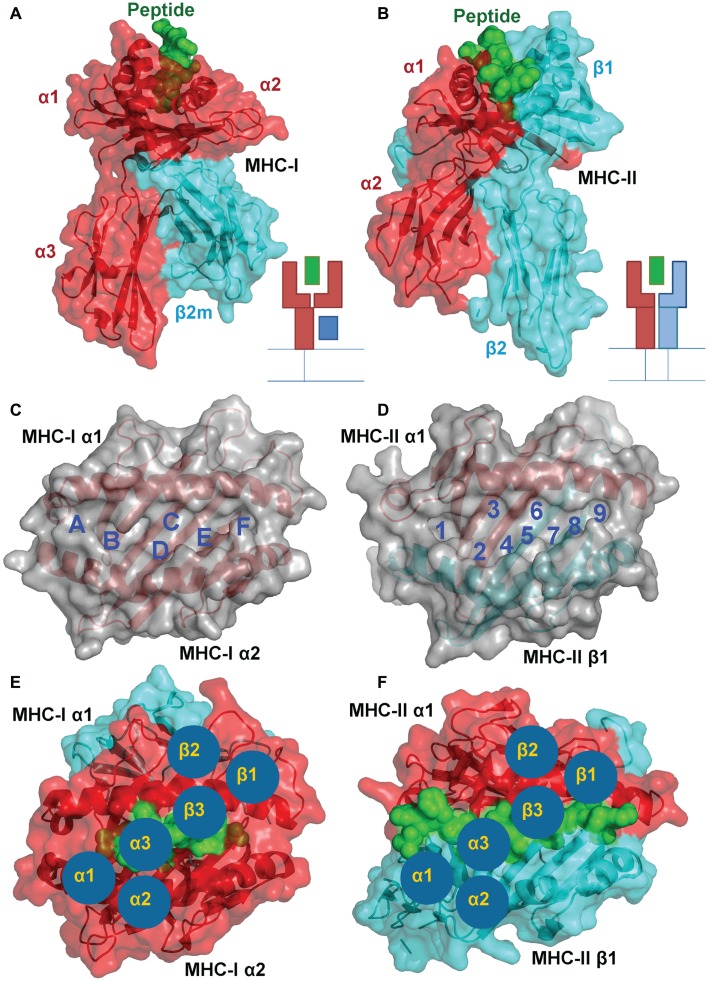
**A structural comparison of pMHC-I and pMHC-II**. Although the subunit compositions of MHC-I (PDB: 1ZHL) **(A)** and MHC-II (PDB: 1KG0) **(B)** are different, the structural conformation they assume is very similar, illustrating their shared role in presenting antigenic peptides (green) to T cells. **(A)** MHC-I is comprised of three α-chain domains (1, 2, and 3 in red) and β2m (cyan), whereas **(B)** MHC-II is comprised of a two domain α-chain (red) and a two domain β-chain (cyan). A top down view of the MHC-I **(C)** and MHC-II **(D)** demonstrates the two molecules form similar peptide binding grooves comprised of two anti-parallel α-helices that form a channel in which the peptide can bind in an extended conformation, and eight anti-parallel β-sheets that provide specific peptide binding pockets in the base of the groove. These pockets are lined with polymorphic residues that define the size and chemical characteristics of each pocket, and therefore the specific peptide binding motif and register that can be accommodated by different MHC alleles. TCR binding to pMHC-I **(E)** and pMHC-II **(F)** is also conserved. The three complementarity determining loops (CDRs) of the TCR (blue circles) bind in a very similar overall orientation with the TCR α-chain over the N-terminus of the peptide and the TCR β-chain over the C-terminus

In spite of the differing subunit compositions of the two MHC classes, they are structurally very similar (Figures 1A,B). The peptide binding groove, in both cases, is comprised of two anti-parallel α-helices that form a channel in which the peptide can bind in an extended conformation, and eight anti-parallel β-sheets that provide specific peptide binding pockets in the base of the groove (Figures 1C,D) (3, 6). Peptides are selected according to their ability to bind to these MHC allele specific pockets within the floor of the peptide binding groove using peptide anchor residues. All of the currently available structural data suggest the TCRs bind to both pMHC-I and II with a fixed polarity (TCRα chain over the N-terminus of the peptide and the TCRβ chain over the C-terminus) and make similar interactions with the bound peptide and MHC surface (Figures 1E,F). Thus, the overall mechanism by which TCRs interact with MHC-I and II to initiate T cell activation is closely matched.

**Figure 2:**
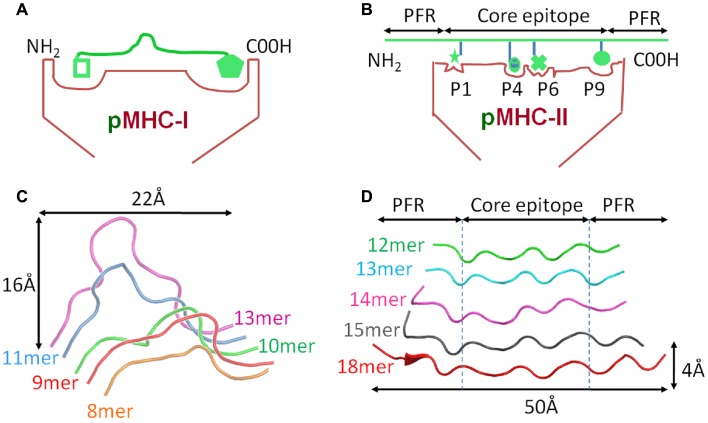
**Comparison of peptide conformations presented by MHC-I and MHC-II**. Cartoon cross sections of the pMHC-I **(A)** and pMHC-II **(B)** binding grooves, show the key anchor sites in the floor of each groove determine which peptide can associate and the conformation it can assume. **(C)** The structural database of pMHC-I complexes shows that peptides presented by a MHC-I molecules (represented as ribbon cartoons) generally assume a central bulged conformation. As peptide length increases, the “closed” nature of the pMHC-I binding groove forces the central residues of the peptide up out of the groove to accommodate the extra residues. **(D)** In contrast, the pMHC-II binding groove is “open” enabling longer peptide to extend out of the groove at form peptide flanking regions. Thus, peptides presented by MHC-II molecules (represented as ribbon cartoons) generally assume a much flatter conformation in the MHC-II binding groove, irrespective of the length of the peptide presented.

Despite these similarities, MHC-I and II present peptides in a distinct manner that is governed by the composition of the MHC peptide binding groove. The closed conformation of the MHC-I α_1_α_2_ binding grove (Figure 2A) restricts peptide length to ∼8–13 amino acids (most commonly 9 or 10mers) (3, 7). In contrast, the MHC-II α_1_β_1_ binding groove comprises an *open-ended* conformation (Figure 2B) that allows variable length peptides to bind. The core binding 9mer contains the motif for binding to the particular MHC-II heterodimer, but eluted and sequenced peptides often reveal families of processed peptides ∼12–20 amino acids (referred to as nested sets) sharing the core binding region (3, 8, 9). MHC-I-restricted peptides usually bind to the MHC surface using anchor residues located at, or near, the N- and C-termini of the peptide. Depending on the length of the peptide, this binding mode squeezes the central peptide residues up so that they extend out the groove (central bulge), exposing peptide side chains for direct interaction with the TCR (3, 10). Longer MHC-I peptides can only be accommodated by forming a larger central bulge, which presumably constrains the length of the peptide beyond a certain threshold (Figure 2C; Table 1).

**Table 1:** **Comparison of peptide conformations presented by MHC-I and MHC-II**.

MHC	Protein	Peptide length (mer)	Peptide Sequence	Height (Å)	Width (Å)	PDB (Ref)
HLA-B*3501	HIV-1 NEF_75–82_	8	VPLRPMTY	4	21	1A1N (56 )
HLA-A*0201	Flu A Matrix_58–66_	9	GILGFVFTL	6	22	1HHI (57 )
HLA-A*0201	MART-1_26–35_	10	EAAGIGILTV	8	22	2GT9 (58 )
HLA-B*3501	EBV_407–417_	11	HPVGEADYFEY	13	22	2FZ3 (59 )
HLA-B*3501	M-CSF_4–17_	14	LPAVVGLSPGEQEY	16	22	1XH3 (60 )
DRB1*0401, DRA1*0101	Collagen II _261–273_	12	AYMRADAAAGGA	4	35	2SEB (61)
DRB1*0101, DRA1*0101	HA_306–318_	13	PKYVKQNTLKLAT	4	37	1DLH (12)
DRB1*1501, DRA1*0101	MBP_85–99_	14	ENPVVHFFKNIVTP	4	42	1BX2 (62)
DRB1*0101, DRA1*0101	MART-1_100–114_	15	APPAYEKLSAEQSPP	4	44	3L6F (63)
DRB1*0101, DRA1*0101	CLIP_102–120_	19	KPVSKMRMATPLLMQALPM	4	50	3PDO (64)

MHC-II restricted peptides contain a central binding motif of nine “core” amino acids that bind to the MHC-II groove via an extensive hydrogen bond network between the MHC-II groove and the peptide backbone (Figure 2B). Peptide side chains also form contacts with allelic specific pockets of the MHC-II binding groove. These pockets, usually P1, P4, P6, and P9, are lined with polymorphic residues that define the size and chemical characteristics of each pocket, and therefore the specific peptide binding motif and register that can be accommodated by different MHC-II alleles (Figure 1D) (11, 12). Amino acids that are outside of the “core” peptide region can extend out of the open MHC-II binding groove forming so called “peptide flanking regions” (PFRs) at both the N- and C-terminus (Figure 2D; Table 1). Thus, although pMHC-I and pMHC-II are similar in their overall structure and function, the nature of peptide presentation is generally distinct (e.g., bulged versus flat peptides). These differences present different challenges for TCR binding at the atomic level. For example, the flat binding surface and lack of a central peptide bulge may enable MHC-II restricted TCRs to adopt a more flexible binding mode compared to MHC-I restricted TCRs. In support of this notion, the structures of a number of TCR-MHC-II complexes have shown that, although the binding mode can be very similar to the classical diagonal TCR-MHC binding mode (13–17), some MHC-II restricted TCR bind with highly unorthodox conformations (18, 19).

## MHC-II Restricted TCRs have Weaker Binding Affinity Compared to pMHC-I Restricted TCRs

**Figure 3:**
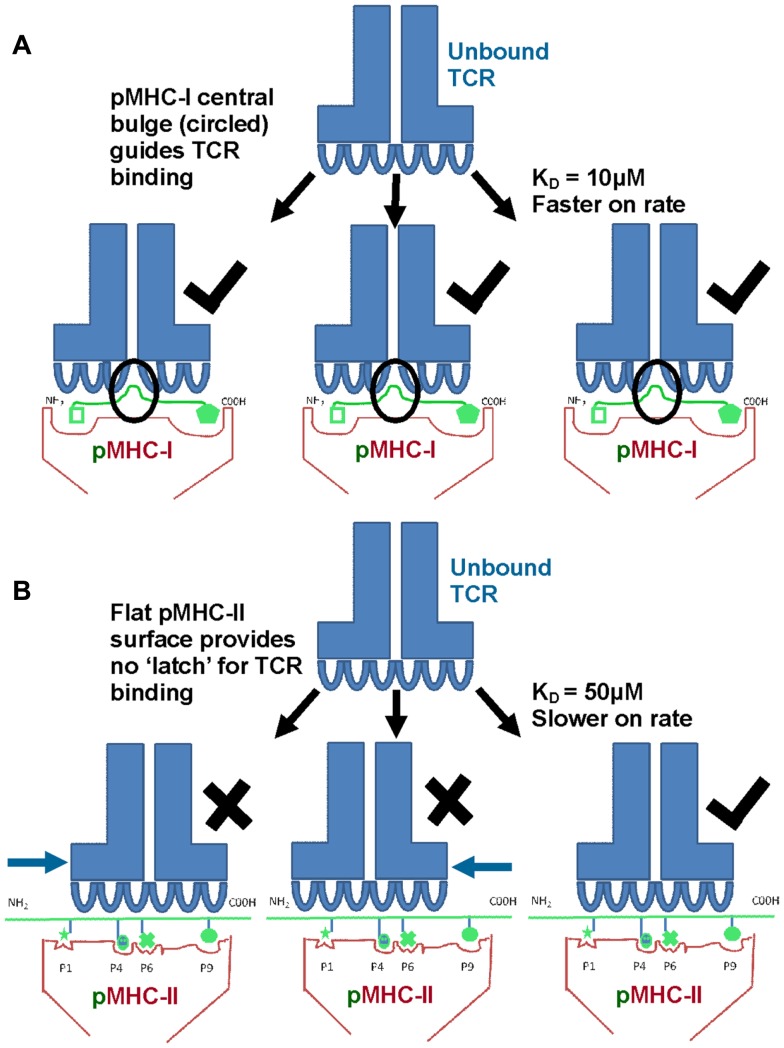
**TCRs bind with stronger affinity to pMHC-I compared to pMHC-II**. Biophysical studies have shown that TCR/pMHC-I binding affinities are, on average, five times stronger compared to equivalent TCR/pMHC-II interactions (i.e., viral pMHC-I restricted TCRs versus viral pMHC-II restricted TCRs) because of faster on-rate for TCR/pMHC-I binding compared to that of TCR/pMHC-II. These differences could be due to the structural differences in peptide presentation between MHC-I and MHC-II. **(A)** Cartoon of TCR binding to pMHC-I. The presence of a solvent exposed central bulge for MHC-I peptide presentation may represent a structurally advantageous feature for TCR binding, providing an anchor point that can guide the TCR into the correct binding orientation to engage its cognate ligand. **(B)** Cartoon of TCR binding to the flatter surface of pMHC-II. This relatively featureless surface provides no dominant structural feature for the TCR to “latch” onto, and may reduce the chance of a productive TCR/pMHC-II interaction occurring (explaining the slower on-rate and weaker affinity compared to TCR/pMHC-I interactions).

Biophysical studies have shown that TCR/pMHC affinity is relatively weak (*K*_D_ = 100 nM–270 μM), with fast kinetics, compared to antibody binding (usually nM–pM affinity) (20, 21). We have recently shown that TCR/pMHC-I binding affinities are, on average, fives times stronger compared to equivalent TCR/pMHC-II interactions (i.e., viral pMHC-I restricted TCRs versus viral pMHC-II restricted TCRs) (Figures 3A,B), which has limited the usefulness of pMHC-II multimers for the identification, isolation and detection of antigen specific CD4^+^ T cells. This distinction in affinity was mainly due to a significantly faster on-rate for TCR/pMHC-I binding compared to that of TCR/pMHC-II, while the off-rate or half-lives of all of the TCR/pMHC interactions were relatively conserved, possibly indicating a more important role for off-rate in determining T cell activation. The consistent difference in binding affinity between TCRs restricted to either pMHC-I or pMHC-II is extraordinary when considering that the same pools of genes, on chromosome 9, encode the human TCR for both types of αβ T cell (22). The TCR itself is expressed before positive selection, at which point immature T cells express both the CD4 and CD8 co-receptors (double positive). Once positively selected, immature thymocytes become single positive for either CD4 or CD8 (22). Until this point, the thymocyte, which has already developed antigen specificity through its TCR, can theoretically have either cell fate (23). Considering these shared genetic and developmental processes, it is possible that the differences in MHC restricted TCR binding is conferred by the variations in the “antigenic landscape” of pMHC-I versus pMHC-II.

As discussed above, peptides presented by MHC-I molecules generally assume a central bulged conformation, often requiring conformational adjustments in the binding regions of the TCR during ligand engagement (Figure 2C) (24–26). An extreme example of this observation is a 13mer Epstein-Barr virus derived peptide presented by HLA-B^∗^3508 which forms a “superbulge” extending nearly 20Å out of the MHC-I binding groove (26). In contrast, MHC-II presented peptides generally assume a much flatter conformation in the MHC-II binding groove (13, 14) (Figure 2D). The presence of a solvent exposed central bulge for MHC-I peptide presentation may represent a structurally advantageous feature for TCR binding, providing an anchor point that can guide the TCR into the correct binding orientation to engage its cognate ligand (Figure 3A). Conversely, the flat, and relatively featureless surface of pMHC-II confers no dominant structural feature for the TCR to “latch” onto, and may reduce the chance of a productive TCR/pMHC-II interaction occurring (explaining the slower on-rate and weaker affinity compared to TCR/pMHC-I interactions) (Figure 3B). This notion is consistent with our recent observation that a MHC-II restricted TCR underwent minimal conformational adjustments during binding compared to most MHC-I restricted TCRs (27). The immunological significance of these topological and biophysical distinctions between MHC-I and MHC-II is still unclear. However, the difference in binding affinity between MHC restricted TCRs may represent a biophysical characteristic that relates to cellular function.

## MHC-II Antigen Processing Generates Variable Length Peptides

The “flat” surface of pMHC-II may contribute to the reduced affinity of MHC-II restricted TCRs. However, a striking difference in the peptides bound to MHC-II is the presence of non-bound PFRs, which may be available to interact with adjacent membrane proteins on the same or different cells. These PFRs can vary in length generating nested sets of peptides that are presented on the surface of APCs (28, 29). One consequence of having a longer PFR is an increased binding affinity of peptides to the MHC-II (30–33), and therefore an increased probability of a meaningful interaction with a cognate T cell.

**Figure 4:**
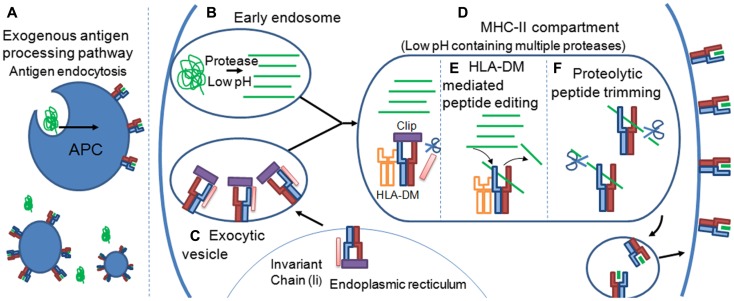
**Peptide flanking regions are determined during the MHC-II antigen processing pathway**. **(A)** Extracellular protein antigens are endocytosed by tissue resident APCs. **(B)** The pH of the endosome containing potential antigens progressively decreases, activating proteases which cleave captured proteins. **(C)** Newly synthesized MHC-II molecules reside in the endoplasmic reticulum (ER) in complex with the MHC-II associated invariant chain (I_i_), which “plugs” the MHC-II binding groove, preventing ER derived peptides from premature peptide association. **(D)** Exocytic vesicles containing precursor Ii:MHC-II complexes then combine with endosomes containing exogenous peptide fragments forming the MHC-II compartment. Formation of the MHC-II compartment results in proteolytic cleavage of the I_i_ chain leaving a 24 amino acid remnant called the class II-associated invariant-chain peptide (CLIP) within the binding groove of the MHC-II molecule. The acidic pH of the MHC-II compartment and presence of the chaperon, HLA-DM, allows peptide exchange between CLIP and high affinity complementary peptides proteolysed in the endosomal compartment. **(E)** Peptide selection, that presumably plays a strong role in determining the characteristics of PFRs, is also facilitated by HLA-DM in a process termed “peptide-editing” which ensures that only stable MHC-II peptide complexes are expressed and transported to the cell surface for potential TCR interactions. **(F)** The final pMHC-II, loaded with exogenous peptide, can also be modified further in a process termed peptide trimming that may play a role in governing PFR length. pMHC-II molecules are then transported to the cell surface for interrogation by CD4^+^ T cells.

These variable PFRs are generated by proteolytic processes during the exogenous antigen processing pathway that has been reviewed in detail elsewhere (34, 35). Briefly, extracellular protein antigens are endocytosed by tissue resident APCs (Figure 4A). The pH of the endosome containing potential antigens progressively decreases, activating proteases which cleave captured proteins (Figure 4B). Newly synthesized MHC-II molecules reside in the endoplasmic reticulum (ER) in complex with a stabilizing chaperon, calnexin. To prevent premature peptide association with the MHC-II binding groove by ER derived proteins, the groove is “plugged” with a protein known as the MHC-II associated invariant chain (I_i_) (36) (Figure 4C). Exocytic vesicles containing precursor Ii:MHC-II complexes then combine with endosomes containing exogenous peptide fragments forming the MHC-II compartment (Figure 4D). The acidic pH of the MHC-II compartment and presence of the chaperon, HLA-DM (37), allows peptide exchange between the class II-associated invariant-chain peptide (CLIP) and high affinity complementary peptides proteolysed in the endosomal compartment. Peptide selection, that presumably plays a strong role in determining the characteristics of PFRs, is also facilitated by HLA-DM in a process termed “peptide-editing” which ensures that only stable MHC-II peptide complexes are expressed and transported to the cell surface for potential TCR interactions (38, 39) (Figure 4E). Structural modeling of HLA-DM association with pMHC-II indicated that peptide editing was achieved through conformational changes around pocket 1 (P1) of the binding groove, a pocket crucial for the stability of the peptide-MHC-II complex (40). Such conformational changes induced by HLA-DM were thought to weaken the hydrogen bond network between the bound peptide and MHC-II molecule and facilitate peptide release (41). A recent co-complex structure of HLA-DM with HLA-DRα^∗^0101; β^∗^0101 (HLA-DR1) has confirmed this experimentally, revealing that HLA-DM binding induced a conformational change in the α-helix of the DRα chain in the peptide binding groove (42). This change enabled two HLA-DR1 residues, DRα phenylalanine 51 and DRβ phenylalanine 89, to bind to, and stabilize, the P1 binding pocket of the MHC-II binding groove, presumably blocking the association of weakly binding peptides. Thus, only the association of high affinity peptides with MHC-II results in displacement of these residues, enabling a revision in the conformation of MHC-II and the dissociation of HLA-DM (40, 42).

It is possible that the proteolytic events that occur before peptide-MHC-II loading govern the final pool of peptides available for selection during MHC-II peptide loading. However, it has also been suggested that the final pMHC-II, loaded with exogenous peptide, can be modified further in a process termed peptide trimming, whereby the length of the PFRs can be edited (Figure 4F) (43, 44). These processes demonstrate a remarkable degree of complexity and control during MHC-II peptide selection that is still not fully understood. The antigen processing by cellular proteases and the generation of pMHC-II may also be influenced by cell extrinsic factors such as inflammatory cytokines, e.g., IFNs (45), as well as cell intrinsic factors reflecting the type/subtype of APC (46, 47). The cellular machinery involved in antigen processing and presentation is different between cell types (48), hence the determinants resulting from protein digestion may vary depending on cell type or subtype (e.g., B cell versus macrophage; CD8^+^ versus CD8^-^ dendritic cells etc); and context (e.g., inflamed versus non-inflamed tissues, anatomical location). It is conceivable that a range of determinants presented in a lymph node may differ from those presented at the primary site of infection in both a quantitative fashion, i.e., the number of pMHC-II complexes per cell, and qualitative fashion i.e., length and type of PFRs and hence offer a local control of CD4^+^ T cell responses accordingly.

## Modulating CD4^+^ T Cell Responses via Altered Peptide Flanking Residues

There is convincing evidence that PFRs can modulate T cell function (49). A study of a HIV-I p24 (GAG) epitope, presented by HLA-DR1, revealed that antigen specific T cell activation was enhanced with longer flanking residues. Structural analyzes showed that the C-terminal flank could form a hairpin turn, raising the possibility that MHC-II PFRs may form more complex conformations that could directly impinge on TCR binding (50). Thus, the open ended nature of the MHC-II binding groove, that allows long peptides to extend beyond the binding region at both the N- and C-terminus (Figures 2 and 3), may play a direct role during T cell antigen recognition. In support of this notion, it has been demonstrated that removal of C-terminus PFRs from the immunodominant epitope in hen egg lysosome_52–61_ (HEL) significantly altered the immunogenicity of the epitope, reducing T cell sensitivity (9).

Our previous work, using sequence analysis of eluted peptide ligands from a range of allelic variants of MHC-II molecules, has identified allele-transcending enrichments in PFRs at the peptide C- and N-terminus (51, 52). These data show that a range of different modifications to PFRs could modulate specific CD4^+^ T cell responses including amino acids with biochemically distinct side chains (52, 53). The identification of these PFR amino acid enrichment patterns suggests that they play a role during CD4^+^ T cell activation and can modulate antigen recognition. Further studies using antigen specific CD4^+^ T cell clones demonstrated that PFR modifications could enhance CD4^+^ T cell activation (53). Although a wide range of different amino acid substitutions in PFRs could generate stronger CD4^+^ T cell responses, we observed that basic residues at the peptide C-terminus, or acidic residues in the N-terminus, were most commonly enriched and generated enhanced CD4^+^ T cell responses across different MHC-II alleles and different peptides. Studies focusing on the C-terminal PFRs, in which the basic amino acid, arginine was substituted into the C-terminal flank (at position 10 or 11) of known T cell epitopes from haemaggluttinin (HA) and myelin basic protein (MBP), demonstrated that these alterations led to a significant increase in CD4^+^ T cell responses (52, 53). Screening T cells which recognized this same MBP-derived epitope with a combinatorial library also revealed a preference for C-terminal basic residues (54).

**Figure 5:**
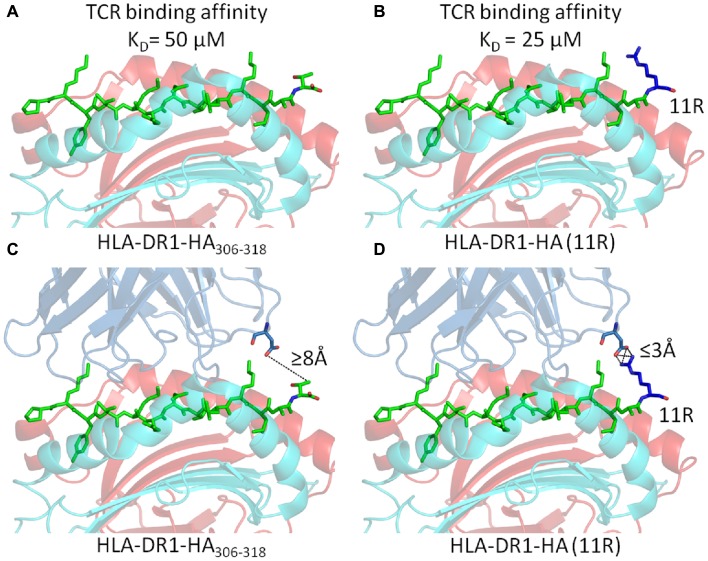
**Substitution of Arginine substitutions in the C-terminal flanking region of the native Flu1 peptide increases binding affinity**. **(A,B)** Substitution of arginine at position 11 (blue) in the HA_305–320_ epitope generates around a twofold increase in TCR binding affinity. **(C,D)** Cartoon representation of the interaction between the TCR and C-terminal PFR (modeled from PDB: 1FYT). **(C)** The TCR β-chain is beyond the limits for atomic contacts with HA_305–320_ P11 (dotted line). **(D)** Modeling shows that a new interaction, possibly a salt bridge, could be formed between the TCR β-chain and arginine (blue) substituted at position 11 of the HA_305–320_ peptide. This new interaction could explain the increase in affinity observed for cognate TCR binding to the HA_305–320_ peptide and HA_11R_.

However, in all of these examples, the mechanism for the effect of PFRs on T cell responsiveness had remained elusive. Two possibilities can be considered. Firstly, PFR modifications may alter the stability of pMHC-II molecules (a notion that has been experimentally observed (30–33), altering their expression levels at the surface of APCs. However, we have demonstrated that, although the substitution of basic residues in the C-terminus increased T cell activation, they actually reduced peptide/MHC binding (52). Secondly, if the TCR can directly contact the PFRs, then modifications in the PFR could alter TCR binding affinity and subsequent T cell activation. In order to investigate the second possibility, we conducted biophysical experiments by surface plasmon resonance using cloned TCRs specific for an influenza epitope (HA_305–320_) presented by HLA-DR1 (53). The substitution of arginine into either position 10 (HA_10R_) or 11 (HA_11R_) of HA_306–318_ generated approximately twofold increase in TCR binding affinity (Figures 5A,B). Intriguingly, analysis of the TCR clonotypic repertoire of peptide-expanded influenza-specific CD4^+^ T cells from HLA-DR1^+^ donors in response to HA_305-320_ or arginine altered variants (HA_10R_ and HA_11R_) demonstrated a marked alteration in TCR usage, with a striking focusing of the response when using the peptides which are known to increase TCR binding i.e., number of clonotypes for HA > HA_10R_ > HA_11R_. The structure of HLA-DR1-HA_306–318_ in complex with the MHC-II restricted TCR, HA1.7, has been solved by X-ray crystallography (55). This structure demonstrated that the TCR could not directly contact the short side chains of either Alanine at P10, or Threonine at P11 in the universal HA_306–318_ epitope (Figure 5C). The closest proximity between the TCR and either P10 or P11 of the HA_306–318_ peptide was over 8Å, which was beyond the limits for atomic contacts. However, structural modeling of the substitution of arginine, which has a long acidic side chain, at either P10 or P11 indicated this gap could be closed allowing additional interactions to form between the peptide and the TCR (Figure 5D) (53). These potential new contacts could offer an explanation for the stronger binding affinity between the HA1.7 TCR and the P10 or P11 substituted DR1-HA_306–318_ epitope.

## Conclusion

The function of classical MHC molecules is presentation of peptide epitopes to the cell mediated arm of adaptive immune response. However, the subtle differences that exist between the two classical forms of MHC, with respect to antigen processing and structural architecture, significantly alters the nature of the peptide each class of MHC can present upon the cell surface. In particular, the closed binding groove of MHC-I forces bound peptides to bulge in the center, compared to the open binding groove of MHC-II that allow PFRs to form. *In vitro* experiments using a variety of antigens in mice and human systems, including HA, GAG, MBP, and HEL, have demonstrated that the PFRs of an epitope can profoundly affect CD4^+^ T cell function. Generation and selection of different PFRs might be governed according to the anatomical location, inflammatory milieu and particular types of APC involved during antigen processing. Thus, a key question that remains is whether particular changes in PFRs occur through a random, stochastic process, or whether changes are purposefully intentioned to control or alter the nature of a specific immune response. Irrespective of the answer to this question, our recent data revealed that experimental PFR modifications could enhance TCR/pMHC-II affinities closer to the range typically observed for TCR/pMHC-I interactions. This exciting observation suggests that augmentation of pMHC-II antigens through C-terminal PFR modifications might be a useful strategy to enhance MHC-II restricted TCR binding affinity and CD4^+^ T cell responsiveness, with attendant implications for vaccination and other immune system interventions.

## Conflict of Interest Statement

The authors declare that the research was conducted in the absence of any commercial or financial relationships that could be construed as a potential conflict of interest.
